# Associations of Sitting Time and Leisure-Time Physical Activity with Mortality Across Cardiovascular–Kidney–Metabolic Syndrome Stages

**DOI:** 10.3390/healthcare14213636

**Published:** 2026-10-27

**Authors:** Fengqing Li, Ying Zhang, Ziyuan Yin, Qingxuan Zeng, Yi Zhang, Yunfeng Liu

**Affiliations:** 1 Department of Endocrinology, First Hospital of Shanxi Medical University, Shanxi Medical University, Taiyuan 030001, China; 2 Department of Pharmacology, School of Basic Medicine, Shanxi Medical University, Taiyuan 030001, China; 3 School of Pharmacy, Shanxi Medical University, Taiyuan 030001, China; 4 Medicinal Basic Research Innovation Center of Chronic Kidney Disease, Ministry of Education, Shanxi Medical University, Taiyuan 030001, China

**Keywords:** cardiovascular–kidney–metabolic syndrome, daily sitting time, leisure-time physical activity, mortality

## Abstract

Background: Cardiovascular–kidney–metabolic (CKM) syndrome confers mortality risk, but whether CKM severity modifies associations of sitting time and leisure-time physical activity (LTPA) with mortality is uncertain. Methods: We examined 15,444 adults aged 20–79 years in the National Health and Nutrition Examination Survey (2007–2018), linked to mortality records through 2019. Survey-weighted multivariable Cox proportional-hazards models were used to estimate hazard ratios (HRs) for all-cause and heart-disease mortality. Results: Over a median of 6.6 years, 1483 deaths occurred, including 354 heart-disease deaths. In survey-weighted multivariable Cox models, higher leisure-time physical activity was associated with lower all-cause and heart-disease mortality across CKM strata. Among participants with advanced CKM, sitting >8 versus ≤4 h/d was associated with higher all-cause mortality (HR, 2.09; 95% CI, 1.61–2.72) and heart-disease mortality (HR, 2.52; 95% CI, 1.62–3.94); however, formal interaction tests did not provide clear evidence of overall effect modification by CKM severity. Participants reporting <150 moderate-equivalent minutes of LTPA per week and >8 h of sitting per day had the largest point estimate for all-cause mortality relative to the reference group. Conclusions: These findings support the consideration of both sitting time and LTPA when assessing adults across the CKM spectrum.

## 1. Introduction

Cardiovascular–kidney–metabolic (CKM) syndrome encompasses the interconnected cardiovascular, kidney and metabolic disorders that collectively contribute to substantial morbidity and premature mortality [1,2]. The American Heart Association CKM framework defines five stages (0–4), spanning optimal cardiometabolic health, excess or dysfunctional adiposity, metabolic risk factors and kidney disease, subclinical cardiovascular disease (CVD) and established CVD [1,3]. This staging system provides a clinically relevant framework in which to examine whether modifiable behaviors have different prognostic implications across the CKM continuum.

Physical activity and sedentary behavior are related but distinct dimensions of daily movement and may coexist within the same individual [4]. Both are associated with cardiometabolic health and mortality [5,6,7,8]. Their joint assessment may therefore provide information beyond either behavior alone, without implying behavioral compensation. Although leisure-time physical activity (LTPA) and sitting time have been associated with mortality in the general population and in people with diabetes or cardiovascular disease [9,10,11,12,13], evidence across the broader CKM spectrum is limited. It remains unclear whether CKM severity modifies their associations with all-cause and heart-disease mortality [14]. This question requires formal interaction testing rather than a comparison of subgroup-specific significance levels.

We therefore used nationally representative National Health and Nutrition Examination Survey (NHANES) data to examine the separate and joint associations of daily sitting time and LTPA with all-cause and heart-disease mortality across CKM stages. We evaluated associations in non-advanced (stages 0–2) and advanced (stages 3–4) CKM and tested CKM by-movement-behavior interaction terms.

## 2. Methods

### 2.1. Study Design and Participants

This prospective cohort study analyzed data from six consecutive cycles of the National Health and Nutrition Examination Survey (NHANES) 2007–2018. The NHANES protocols were approved by the National Center for Health Statistics Ethics Review Board, and written informed consent was obtained from all participants. Participants were restricted to adults aged 20–79 years because age is top-coded at 80 years in the public-use NHANES data, limiting precise age-dependent risk estimation among older participants.

**Figure 1:**
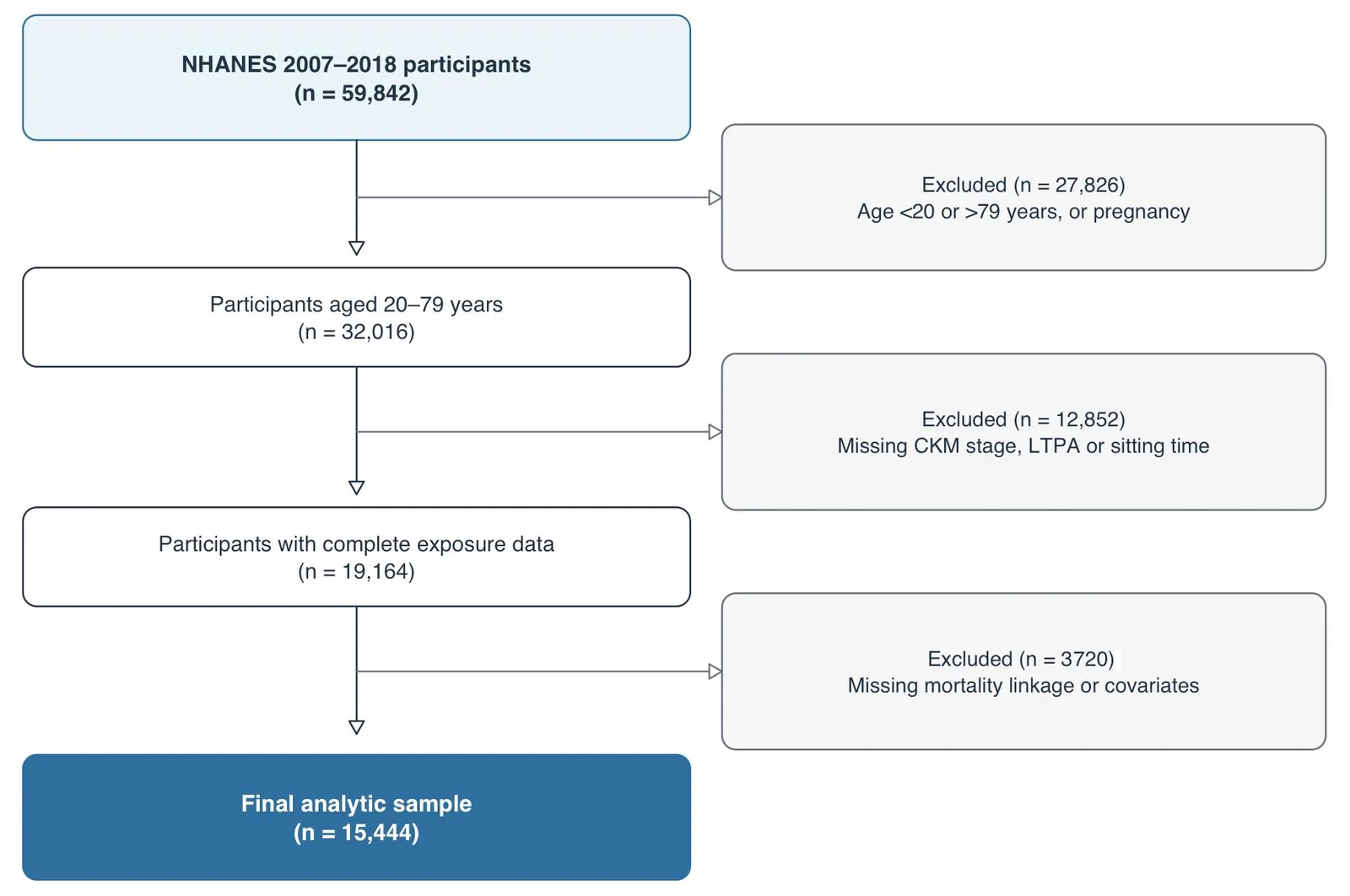
Flowchart of participant selection in NHANES 2007–2018.

Among 59,842 participants from NHANES 2007–2018, 27,826 were excluded because they were outside the age range of 20–79 years or pregnant, 12,852 because of insufficient information to determine CKM status or missing LTPA or sitting-time data, and 3720 because of missing mortality linkage or prespecified covariates, leaving 15,444 participants for the primary analysis (Figure 1).

### 2.2. CKM Syndrome Classification

CKM syndrome stages were classified according to the 2023 American Heart Association Presidential Advisory Statement into five stages (0–4) [1]. For the primary analysis, participants were grouped as non-advanced CKM (stages 0–2) and advanced CKM (stages 3–4) [15]. This grouping was used to provide clinically interpretable estimates with adequate precision and does not imply homogeneous risk within either stratum. Stage assignment was operationalized using prespecified criteria available in NHANES. Detailed operational definitions, including the approach to stage 3 in the absence of consistently available subclinical CVD measures, are provided in Supplementary Table S1.

### 2.3. Physical Activity and Sedentary Behavior Assessment

LTPA and daily sitting time were assessed using standardized NHANES physical activity questionnaires. LTPA was calculated as weekly minutes of moderate-intensity activity + 2 × weekly minutes of vigorous-intensity activity and is expressed as moderate-intensity-equivalent minutes per week [16]. Separate LTPA analyses used inactive (0 min/wk), insufficiently active (>0 to <150 min/wk), and active (≥150 min/wk) categories [7,17]. The survey-weighted 33.3rd and 66.7th percentiles of daily sitting time corresponded to 4 and 8 h/d, respectively; sitting time was therefore categorized as ≤4, >4 to ≤8, and >8 h/d. Because reported sitting time was heaped at 4 and 8 h/d, the weighted group sizes were not equal.

### 2.4. Covariates

Sociodemographic variables included sex, age, race/ethnicity (Mexican American, non-Hispanic White, non-Hispanic Black, other Hispanic, other race), poverty-to-income ratio (PIR; total family income divided by federal poverty threshold, categorized as ≤1.39, >1.39 to ≤3.49, >3.49), marital status (married/cohabiting, never married, widowed/divorced/separated), and education (above high school, high school or equivalent, and under high school). Personal behavioral variables included smoking status (never smoker, former smoker, current smoker) and alcohol consumption (never, former, mild, moderate, heavy). Covariates were selected as potential demographic, socioeconomic, and behavioral confounders. Clinical characteristics that directly contributed to CKM stage classification were not routinely included in the primary models to avoid conditioning on components of the stratifying variable and potential overadjustment.

### 2.5. Mortality Ascertainment

The primary outcome was all-cause mortality. Heart-disease mortality was a secondary outcome and was defined as deaths with ICD-10 codes I00–I09, I11, I13, and I20–I51 as the underlying cause of death. Mortality data were obtained from the National Center for Health Statistics Linked Mortality Files, which link NHANES participants to the National Death Index, with follow-up available through 31 December 2019. Follow-up time was calculated from the examination date to the date of death or 31 December 2019, whichever occurred first. For cause-specific Cox analyses of heart-disease mortality, deaths from other causes were censored at the time of death. For graphical analyses, non-heart-disease deaths were treated as competing events when estimating cumulative heart-disease incidence.

### 2.6. Statistical Analysis

All analyses incorporated NHANES sampling weights and accounted for the complex survey design using sampling strata and primary sampling units. Analyses were conducted using R software (version 4.3.1). Baseline characteristics are summarized using weighted proportions for categorical variables and survey-weighted means with weighted standard deviations for continuous variables. For descriptive comparisons across LTPA categories in Supplementary Table S2, continuous variables were compared using survey-weighted linear models with design-based Wald tests, and categorical variables using Rao–Scott design-adjusted F tests.

Survey-weighted Cox proportional-hazards models were used to evaluate sitting time and LTPA separately. In the primary separate-exposure models, sitting time and LTPA were not mutually adjusted. Models were adjusted for age, sex, race/ethnicity, education, PIR, marital status, smoking status and alcohol consumption. The proportional-hazards assumption was evaluated using Schoenfeld residuals, and additional piecewise Cox analyses were performed when evidence of non-proportionality was identified.

To examine the combined associations of sitting time and physical activity with mortality, participants were cross-classified according to daily sitting time (≤4, >4 to ≤8, and >8 h/d) and LTPA-categorized using the guideline-recommended threshold (<150 and ≥150 moderate-equivalent min/wk), resulting in six joint exposure groups. This categorization was used to reflect the guideline-recommended LTPA threshold and to facilitate interpretation of the joint associations. The reference group was defined as participants with ≤4 h/d sitting time and ≥150 moderate-equivalent min/wk LTPA. Hazard ratios were estimated using progressively adjusted survey-weighted Cox models: Model 1 was unadjusted; Model 2 adjusted for age, sex, race/ethnicity, education, PIR, and marital status; and Model 3 further adjusted for smoking status and alcohol consumption.

Formal interaction analyses were conducted in the fully adjusted survey-weighted Cox models. Omnibus interactions were evaluated using design-based Wald tests for CKM stratum × sitting-time category, CKM stratum × LTPA category, joint six-category exposure × CKM stratum, and sitting-time category × three-level LTPA category. Sitting time × LTPA interactions were evaluated in the overall sample and separately within non-advanced and advanced CKM strata.

As a sensitivity analysis, the primary six-category joint exposure was expanded to a nine-category exposure by crossing the three LTPA categories (inactive, insufficiently active, and active) with the three sitting-time categories. Additional sensitivity analyses retained stages 0–2 as a combined stratum while evaluating stages 3 and 4 separately to assess potential heterogeneity within advanced CKM. As an additional sensitivity analysis, sitting-time models were further adjusted for LTPA, and LTPA models were further adjusted for sitting time.

The primary analyses used complete cases with available information on CKM stage, sitting time, LTPA, mortality linkage, and all prespecified covariates. Variable-specific and overlapping missingness patterns were described, and, among age-eligible nonpregnant participants, those included in the complete-case analysis were compared with those excluded because of missing data using the NHANES complex survey design. No imputation was used in the primary analyses. All statistical tests were two-sided, and *p* < 0.05 was considered statistically significant.

## 3. Results

### 3.1. Participant Characteristics

The analysis included 15,444 adults across the CKM spectrum (49.5% women; weighted mean age, 50.0 years). Overall, 68.8% were non-Hispanic White, 23.4% had a PIR of ≤1.39 m, and 15.5% had not completed high school. In addition, 52.7% had never smoked and 38.1% reported mild alcohol consumption (Table 1).

The survey-weighted proportions of participants reporting ≤4, >4 to ≤8 and >8 h per day of sitting were 35.6%, 40.4% and 24.0%, respectively (Table 1). Participants reporting longer sitting time generally had higher educational attainment and household income. For LTPA, 48.2% were inactive, 16.7% were insufficiently active and 35.1% met the recommended level of ≥150 min per week. Participants meeting this recommendation were generally younger and more likely to be men and to have higher educational attainment and household income (Table S2). Participant counts by CKM stage and sex are provided in Table S3. Variable-specific and overlapping missingness patterns are summarized in Table S4. The baseline characteristics of participants included in and excluded from the complete-case analysis are presented in Table S5; the two groups differed in several sociodemographic and behavioral characteristics.

### 3.2. Associations of LTPA and Sitting Time with Mortality

**Figure 2:**
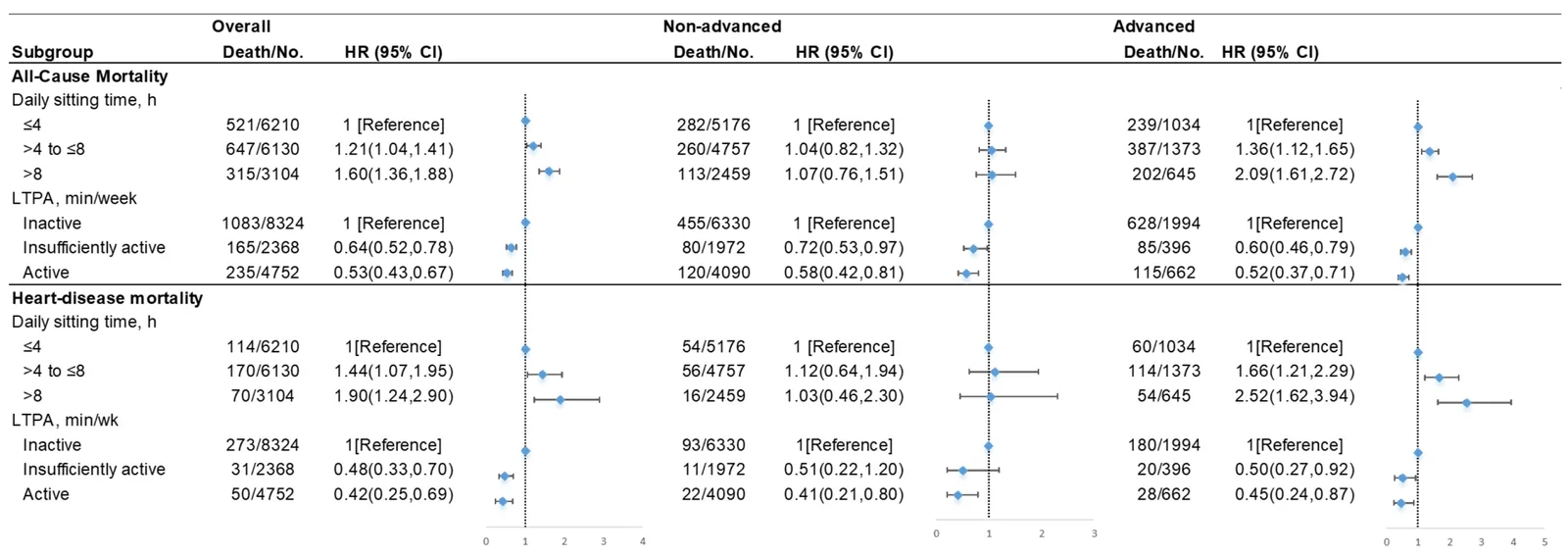
Associations of daily sitting time and leisure-time physical activity (LTPA) with mortality across CKM strata. Fully adjusted models included age, sex, race/ethnicity, education, poverty-to-income ratio, marital status, smoking status, and alcohol consumption. Reference groups were daily sitting time ≤4 h/d and inactive LTPA, respectively. The dotted line indicates HR = 1.0. HR, hazard ratio; CKM, cardiovascular–kidney–metabolic; LTPA, leisure-time physical activity.

During a median follow-up of 6.6 years, 1483 participants died, including 354 from heart disease. In fully adjusted models, higher LTPA was associated with lower all-cause and heart-disease mortality across CKM strata (Figure 2). For sitting >8 versus ≤4 h/d, larger point estimates were observed among participants with advanced CKM (all-cause mortality: HR, 2.09; 95% CI, 1.61–2.72; heart-disease mortality: HR, 2.52; 95% CI, 1.62–3.94). However, omnibus CKM × sitting-time and CKM × LTPA interaction tests did not provide clear evidence of effect modification by CKM stratum (Table S6).

### 3.3. Joint Associations of LTPA and Sitting Time with Mortality

**Figure 3:**
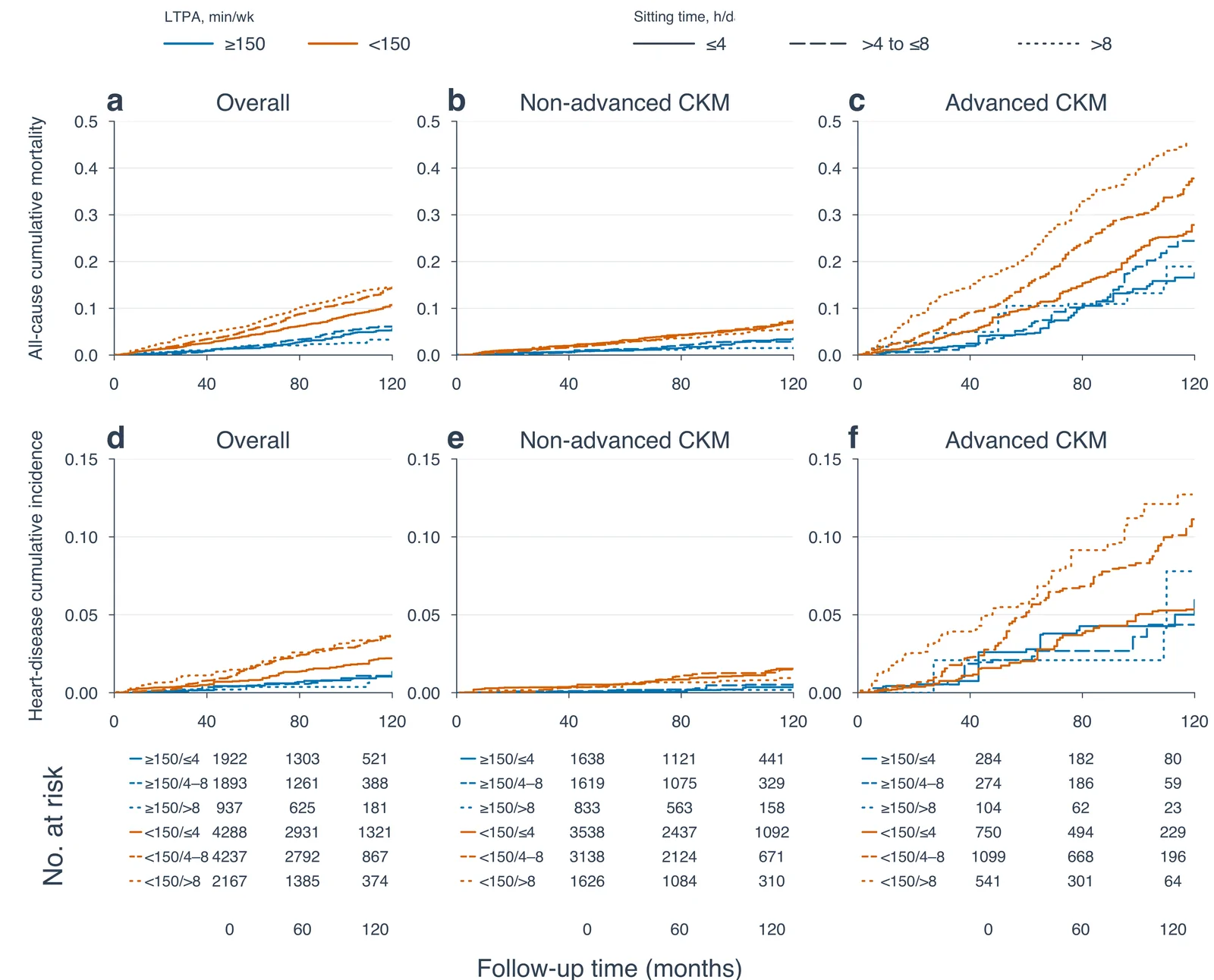
All-cause cumulative mortality and heart-disease cumulative incidence across six joint categories of sitting time and LTPA. Panels (**a**–**c**) show all-cause cumulative mortality, and panels (**d**–**f**) show heart-disease cumulative incidence. For heart-disease mortality, non-heart-disease deaths were treated as competing events. Unweighted numbers at risk are shown at 0, 60, and 120 months. LTPA was categorized as <150 or ≥150 moderate-equivalent min/wk, and sitting time was categorized as ≤4, >4 to ≤8, and >8 h/d. CKM, cardiovascular–kidney–metabolic.

**Figure 4:**
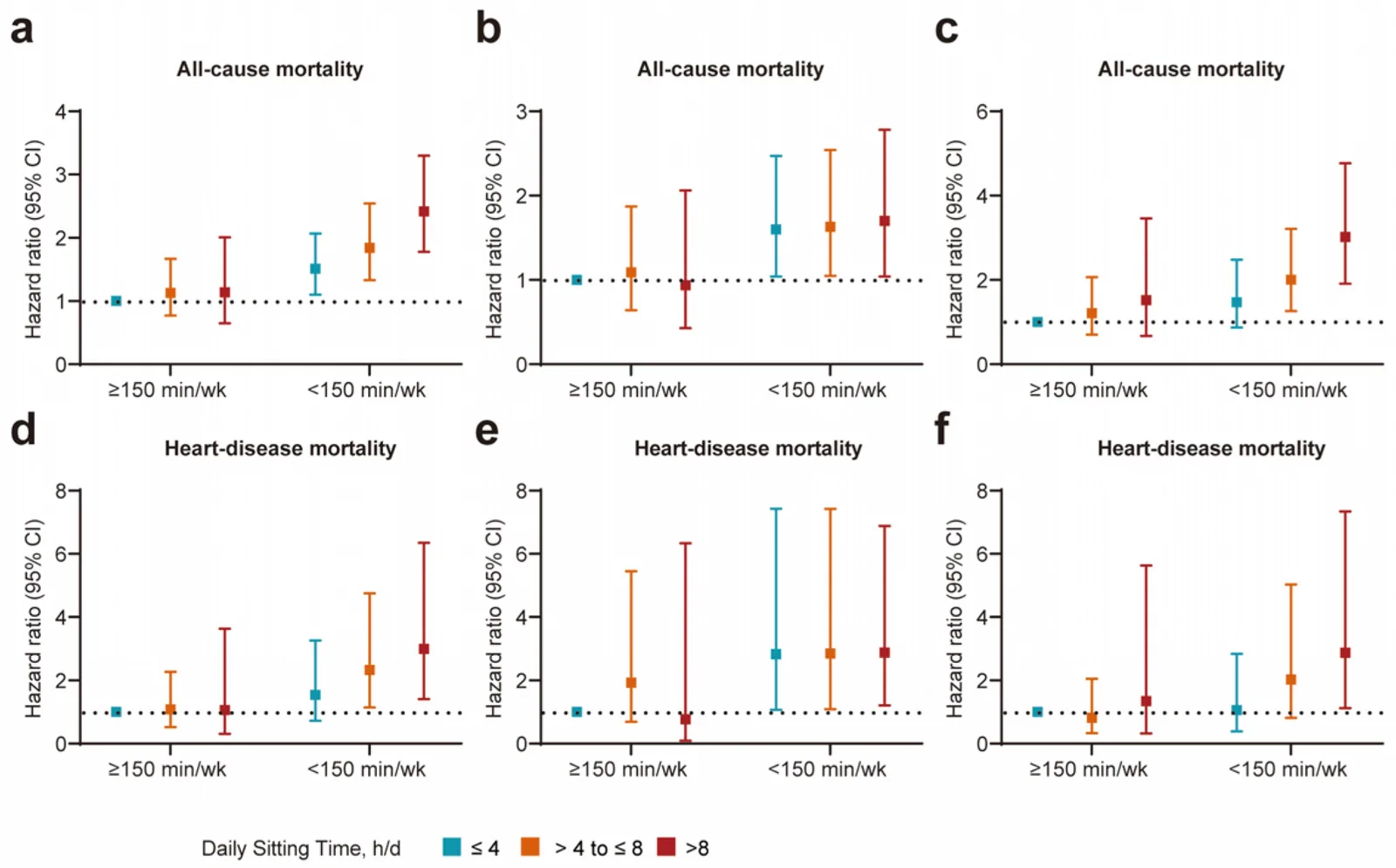
Joint associations of daily sitting time and leisure-time physical activity (LTPA) with mortality by CKM stratum. Panels (**a**–**f**) present results for the overall CKM population (**a**,**d**), non-advanced CKM (**b**,**e**), and advanced CKM (**c**,**f**), respectively. The reference group was LTPA ≥150 min/wk and daily sitting time ≤4 h/d. Models were adjusted for age, sex, race/ethnicity, education, poverty-to-income ratio, marital status, smoking status, and alcohol consumption. CKM, cardiovascular–kidney–metabolic; LTPA, leisure-time physical activity.

In the primary joint analysis, LTPA was categorized as <150 versus ≥150 moderate-equivalent min/wk and combined with the three sitting-time categories, resulting in six joint exposure groups. The weighted prevalence of the joint category defined by LTPA <150 min per week and sitting >8 h per day was 16.0% overall, 15.4% in non-advanced CKM and 18.9% in advanced CKM (Figure S1). Figure 3 shows the all-cause cumulative mortality and heart-disease cumulative incidence across the six joint exposure categories in the overall population and within each CKM stratum. Compared with participants reporting LTPA ≥150 min per week and sitting ≤4 h per day, those reporting LTPA <150 min per week and sitting >8 h per day had the largest point estimate for all-cause mortality relative to the reference group, with HRs of 2.42 (95% CI, 1.78–3.30) overall, 1.70 (95% CI, 1.04–2.78) in non-advanced CKM and 3.02 (95% CI, 1.91–4.77) in advanced CKM. Heart-disease mortality estimates for the corresponding joint categories are presented in Table 2 and Figure 4. Several of these estimates were based on small numbers of events and had wide confidence intervals; these findings were therefore considered exploratory. Formal interaction analyses did not provide clear evidence of interactions between the six-category joint exposure and CKM stratum or between sitting time and LTPA for either mortality outcome (Table S6).

### 3.4. Sensitivity and Stratified Analyses

Sex- and age-stratified analyses showed broadly similar patterns for all-cause mortality (Figure S2). These findings were interpreted descriptively because formal interaction tests for age and sex were not performed. LTPA-stratified analyses showed variation in sitting-related point estimates across activity categories (Figure S3); however, formal sitting time × LTPA interaction tests did not provide clear evidence of effect modification (Table S6). Stage-specific sensitivity analyses showed elevated point estimates for prolonged sitting in both CKM stage 3 and stage 4, while higher LTPA categories generally showed lower mortality point estimates (Table S7). The nine-category joint exposure analysis showed broadly similar all-cause mortality patterns to the primary six-category analysis, whereas several heart-disease mortality estimates were imprecise because of sparse events (Table S8). Results were also generally consistent when sitting-time models were additionally adjusted for LTPA and when LTPA models were additionally adjusted for sitting time (Table S9). Evidence of non-proportional hazards was observed for sitting time and LTPA in some all-cause mortality models. Piecewise Cox analyses showed that the magnitude of the associations varied over follow-up, while the direction of the associations remained generally consistent.

## 4. Discussion

### 4.1. Principal Findings

In this cohort of US adults across the CKM spectrum, higher LTPA was associated with lower all-cause and heart-disease mortality across CKM strata, whereas prolonged sitting showed larger mortality-related point estimates among participants with advanced CKM. However, formal interaction analyses did not provide clear evidence of effect modification by CKM severity or of an interaction between sitting time and LTPA. In the joint exposure analysis, the combination of LTPA below the recommended level and prolonged sitting showed the largest point estimate for all-cause mortality relative to the reference group. These findings characterize the joint associations of sitting time and LTPA with mortality but should not be interpreted as evidence of behavioral compensation, substitution, or causal interaction.

### 4.2. Sitting Time, CKM Severity, and Previous Evidence

The association between prolonged sitting and mortality showed larger point estimates among participants with advanced CKM. Stage-specific sensitivity analyses showed elevated point estimates for sitting >8 versus ≤4 h/d in both CKM stage 3 and stage 4, suggesting that the observed pattern was not confined to stage 4. However, the omnibus CKM × sitting-time interaction did not provide clear evidence of effect modification, and differences between CKM strata should therefore be interpreted descriptively rather than as definitive evidence of effect modification. Recent studies have reported that prolonged sitting is associated with higher all-cause mortality and adverse kidney-function profiles [18], while more advanced CKM stages are associated with greater mortality risk [19]. Greater disease burden, lower physiological reserve, frailty, and functional limitations among individuals with advanced CKM may also contribute to these patterns through residual confounding or reverse causation [20]. Sedentary behavior has been linked to adverse cardiovascular and metabolic outcomes through multiple physiological pathways [5,6].

These findings are broadly consistent with previous evidence linking prolonged sedentary time with higher all-cause and cardiovascular mortality [8,21]. Patterson et al. reported dose–response associations between sedentary behavior and all-cause and cardiovascular mortality, while a large harmonized meta-analysis showed that the mortality association of prolonged sitting varied according to physical activity level [8,21]. Among US adults with diabetes, Dai and Albrecht similarly found that sitting-related all-cause and heart-disease mortality associations differed according to physical activity level [13]. In the present study, however, formal sitting time × LTPA interaction analyses did not provide clear evidence of effect modification across the broader CKM population.

Evidence from populations with kidney disease also supports the relevance of movement behaviors to prognosis. Previous NHANES studies have reported associations of physical activity with mortality among adults with CKD [22,23]. These findings provide context for the present results but do not establish that CKM severity itself modifies the association between prolonged sitting and mortality.

### 4.3. LTPA Across the CKM Spectrum

Higher LTPA was generally associated with lower mortality across CKM strata in the present study. Recent NHANES evidence has similarly reported inverse associations between physical activity and mortality across CKM stages, including recreational physical activity [24]. This finding is consistent with prospective and accelerometer-based studies showing inverse dose–response associations between physical activity and mortality [25], as well as with observations in adults with CKD [22]. A harmonized meta-analysis using accelerometer-measured activity similarly found lower all-cause mortality with higher levels of physical activity [26]. Current physical activity guidelines recommend at least 150–300 min/week of moderate-intensity aerobic activity, or an equivalent combination of moderate- and vigorous-intensity activity, for adults, including those with chronic conditions [7]. Inverse LTPA–mortality associations were observed in both CKM strata, although formal CKM × LTPA interaction tests did not provide clear evidence that these associations differed by CKM severity.

Potential pathways linking higher LTPA with lower mortality include improved insulin sensitivity and metabolic function [27,28,29], higher cardiorespiratory fitness [30], and the anti-inflammatory effects of regular exercise [31]. Regular exercise also induces adaptations across skeletal muscle, adipose tissue, the vasculature, and other metabolic organs that may improve systemic metabolic health [27]. However, these mechanisms were not measured in the present study and should therefore be considered biologically plausible hypotheses rather than demonstrated mediators.

### 4.4. Joint Interpretation of Movement Behaviors

Considering sitting time and LTPA jointly, participants reporting LTPA <150 min/week and sitting >8 h/day showed the largest point estimate for all-cause mortality relative to those reporting LTPA ≥150 min/week and sitting ≤4 h/day. This pattern is broadly compatible with previous evidence showing that mortality associated with prolonged sitting varies according to physical activity level [8,13,32]. More recent prospective studies have likewise linked combinations of greater sedentary time and lower physical activity with higher all-cause mortality [33,34,35], while device-based evidence has also linked prolonged sedentary behavior with cardiovascular outcomes, including cardiovascular mortality [36]. However, formal joint exposure category × CKM stratum and sitting time × LTPA interaction tests in the present study did not provide clear evidence of effect modification (Supplementary Table S6). The joint findings should therefore be interpreted as associations across combinations of sitting time and LTPA rather than evidence that higher LTPA offsets the mortality risk associated with prolonged sitting or that one behavior causally compensates for the other.

### 4.5. Clinical Implications

These findings suggest that both sitting time and LTPA may be relevant when evaluating lifestyle behaviors across the CKM spectrum. Current guidelines recommend regular physical activity and limiting sedentary time among adults, including those living with chronic conditions [7,17]. More recently, the 2026 AHA/ACC/ADA/ASN CKM guidelines emphasized lifestyle modification as an integral component of risk-factor management across the CKM spectrum [37]. Lifestyle interventions have also been emphasized as an important component of comprehensive management across CKM stages [38]. Within this framework, opportunities to reduce prolonged sitting and increase feasible physical activity may be considered while accounting for cardiovascular, kidney, metabolic, and functional status. However, the present observational findings do not establish that modifying either behavior reduces mortality, and interventional studies are needed before specific movement prescriptions can be recommended for mortality reduction. Whether intervention effects differ according to CKM stage also requires prospective evaluation. More broadly, lifestyle interventions may form part of multidisciplinary cardiovascular prevention strategies, particularly in individuals with overlapping cardiovascular, metabolic, and psychosocial risk factors [39].

### 4.6. Strengths and Limitations

This study has several strengths. It included a large sample drawn from the nationally representative NHANES, used long-term mortality linkage, and accounted for the complex survey design. Sitting time and LTPA were evaluated both separately and jointly across clinically relevant CKM strata, and formal interaction analyses were performed rather than relying solely on comparisons of stratum-specific estimates. Stage-specific, joint exposure, and mutual-adjustment sensitivity analyses further allowed the robustness of the main findings to be examined.

Several limitations should also be considered. First, sitting time and LTPA were self-reported at a single examination and were therefore susceptible to recall error, social-desirability bias, and exposure misclassification; changes in behavior during follow-up were not captured. LTPA also did not capture occupational or transportation-related physical activity, so participants with low LTPA may nevertheless have accumulated substantial activity in other domains. Second, residual confounding and reverse causation remain possible, particularly because disease severity, frailty, and functional limitations may influence both sitting time and physical activity. These sources of bias cannot be fully excluded in an observational study. Third, the primary analyses used complete cases. Differences between included and excluded participants indicate the potential for selection bias, and the original NHANES weights do not necessarily account for selection introduced by complete-case restriction. Fourth, CKM stage was operationalized using measurements available in NHANES, and some aspects of the AHA CKM framework, particularly subclinical cardiovascular disease measures relevant to stage 3, could not be fully captured; stage misclassification is therefore possible. In addition, dichotomizing CKM stages into non-advanced and advanced groups may obscure stage-specific heterogeneity and result in some loss of clinical information. Fifth, heart-disease mortality and some subgroup analyses were based on relatively small numbers of events, resulting in wide confidence intervals, and multiple comparisons increase the possibility of chance findings. The proportional-hazards assumption was not fully satisfied in some all-cause mortality models, indicating that the magnitude of some associations may vary over follow-up, although time-stratified analyses showed generally consistent directions of association. Finally, the observational design precludes causal inference, and the findings may not generalize beyond non-institutionalized US adults.

## 5. Conclusions

In conclusion, higher LTPA was associated with lower all-cause and heart-disease mortality across CKM strata, whereas larger mortality-related point estimates for prolonged sitting were observed among participants with advanced CKM; however, formal interaction tests did not provide clear evidence of effect modification by CKM severity. Participants reporting both LTPA <150 min/wk and sitting >8 h/d showed the largest point estimate for all-cause mortality relative to the reference group. These findings support consideration of both sitting time and leisure-time physical activity when evaluating lifestyle behaviors across the CKM spectrum. Whether interventions targeting these behaviors reduce mortality or have differential effects according to CKM stage requires prospective testing.

## Figures and Tables

**Table 1:** Baseline characteristics of study participants according to daily sitting time.

Characteristic	Overall (*n* = 15,444)	≤4 h/d (*n* = 6210)	>4 to ≤8 h/d (*n* = 6130)	>8 h/d (*n* = 3104)
Sex, *n* (weighted %)				
Female	7683 (49.5)	3062 (48.7)	3076 (51.1)	1545 (48.3)
Male	7761 (50.5)	3148 (51.3)	3054 (48.9)	1559 (51.7)
Age, years, weighted mean (SD)	50.0 (15.5)	49.7 (15.5)	50.8 (15.9)	49.4 (14.8)
Race and ethnicity, *n* (weighted %)				
Mexican American	2322 (7.9)	1351 (12.4)	709 (6.2)	262 (4.2)
Non-Hispanic White	6402 (68.8)	2167 (61.7)	2706 (70.5)	1529 (76.3)
Non-Hispanic Black	3423 (10.9)	1280 (11.3)	1416 (11.1)	727 (10.1)
Other Hispanic	1574 (5.1)	805 (7.2)	554 (4.4)	215 (3.3)
Other race and ethnicity	1723 (7.2)	607 (7.3)	745 (7.9)	371 (6.2)
Poverty-to-income ratio, *n* (weighted %)				
≤1.39	5438 (23.4)	2489 (28.4)	2125 (22.9)	824 (16.7)
>1.39 to ≤3.49	5325 (33.1)	2292 (36.6)	2104 (33.3)	929 (27.4)
>3.49	4681 (43.5)	1429 (35.0)	1901 (43.7)	1351 (55.9)
Marital status, *n* (weighted %)				
Married or cohabiting	9332 (65.1)	3892 (66.8)	3636 (64.8)	1804 (63.2)
Never married	2402 (15.1)	847 (13.4)	1016 (15.9)	539 (16.4)
Widowed, divorced or separated	3710 (19.8)	1471 (19.9)	1478 (19.3)	761 (20.5)
Education, *n* (weighted %)				
Above high school	8083 (61.0)	2625 (50.2)	3381 (63.1)	2077 (73.3)
High school or equivalent	3559 (23.5)	1519 (28.0)	1426 (23.0)	614 (17.9)
Below high school	3802 (15.5)	2066 (21.8)	1323 (13.9)	413 (8.7)
Smoking status, *n* (weighted %)				
Never	8091 (52.7)	3358 (51.9)	3119 (52.5)	1614 (54.0)
Former	4123 (27.8)	1572 (26.6)	1678 (27.7)	873 (29.6)
Current	3230 (19.6)	1280 (21.5)	1333 (19.8)	617 (16.4)
Alcohol consumption, *n* (weighted %)				
Never	2080 (9.9)	999 (11.9)	738 (9.0)	343 (8.5)
Former	2658 (13.9)	1034 (13.7)	1140 (15.2)	484 (12.3)
Mild	5333 (38.1)	1947 (34.5)	2216 (39.9)	1170 (40.6)
Moderate	2361 (17.7)	908 (16.9)	887 (16.7)	566 (20.7)
Heavy	3012 (20.3)	1322 (23.1)	1149 (19.2)	541 (17.9)
Leisure-time physical activity, *n* (weighted %)				
Inactive (0 min/wk)	8324 (48.2)	3387 (48.5)	3279 (48.1)	1658 (48.0)
Insufficiently active (>0 to <150 min/wk)	2368 (16.7)	901 (15.7)	958 (16.3)	509 (18.8)
Active (≥150 min/wk)	4752 (35.1)	1922 (35.8)	1893 (35.6)	937 (33.2)
CKM stage, *n* (weighted %)				
Non-advanced (stages 0–2)	12,392 (82.0)	5176 (83.8)	4757 (80.0)	2459 (82.6)
Advanced (stages 3–4)	3052 (18.0)	1034 (16.2)	1373 (20.0)	645 (17.4)

Values are unweighted n (survey-weighted %) unless otherwise indicated. Continuous variables are presented as survey-weighted mean (SD). LTPA is expressed as moderate-intensity-equivalent minutes per week. CKM, cardiovascular–kidney–metabolic; LTPA, leisure-time physical activity; SD, standard deviation.

**Table 2:** Joint associations of daily sitting time and leisure-time physical activity (LTPA) with mortality by CKM stratum.

Outcome	LTPA, Min/Week	Sitting, h/d	Non-Advanced CKM (Stages 0–2)		Advanced CKM (Stages 3–4)	
			Deaths/N	Adjusted HR (95% CI)	Deaths/N	Adjusted HR (95% CI)
All-cause mortality	≥150	≤4	51/1638	1.00 (reference)	47/284	1.00 (reference)
	≥150	>4 to ≤8	56/1619	1.09 (0.64–1.87)	54/274	1.21 (0.70–2.07)
	≥150	>8	13/833	0.94 (0.43–2.06)	14/104	1.52 (0.67–3.46)
	<150	≤4	231/3538	1.60 (1.04–2.47)	192/750	1.47 (0.87–2.48)
	<150	>4 to ≤8	204/3138	1.63 (1.05–2.54)	333/1099	2.01 (1.26–3.21)
	<150	>8	100/1626	1.70 (1.04–2.78)	188/541	3.02 (1.91–4.77)
Heart-disease mortality	≥150	≤4	8/1638	1.00 (reference)	13/284	1.00 (reference)
	≥150	>4 to ≤8	13/1619	1.93 (0.69–5.45)	12/274	0.82 (0.33–2.05)
	≥150	>8	1/833	0.77 (0.09–6.34)	3/104	1.34 (0.32–5.63)
	<150	≤4	46/3538	2.83 (1.07–7.43)	47/750	1.06 (0.39–2.84)
	<150	>4 to ≤8	43/3138	2.85 (1.09–7.42)	102/1099	2.03 (0.82–5.03)
	<150	>8	15/1626	2.88 (1.21–6.88)	51/541	2.87 (1.12–7.34)

Hazard ratios were estimated using survey-weighted Cox proportional-hazards models adjusted for age, sex, race/ethnicity, education, poverty-to-income ratio, marital status, smoking status, and alcohol consumption. The reference group was LTPA ≥150 min/wk and daily sitting time ≤4 h/d. CI, confidence interval; CKM, cardiovascular–kidney–metabolic; HR, hazard ratio; LTPA, leisure-time physical activity. In the primary joint analysis, LTPA was categorized as <150 versus ≥150 moderate-equivalent min/week.

## Data Availability

The datasets from the National Health and Nutrition Examination Survey (NHANES) utilized in this study are publicly accessible via the following website: https://www.cdc.gov/nchs/nhanes/ (accessed on 1 June 2026).

## Supplementary Materials

The following supporting information can be downloaded at: https://www.mdpi.com/article/10.3390/healthcare14213636/s1. Figure S1: Weighted distribution of joint categories of leisure-time physical activity and daily sitting time among individuals with CKM; Figure S2: Sex- and age-stratified associations of leisure-time physical activity and sitting time with all-cause mortality; Figure S3: Associations of daily sitting time with mortality according to leisure-time physical activity among individuals with CKM; Table S1: Operationalization of cardiovascular–kidney–metabolic stages in NHANES 2007–2018; Table S2: Characteristics of the study population by leisure-time physical activity level; Table S3: Participants and all-cause deaths by CKM stage and sex, NHANES 2007–2018; Table S4: Variable-specific and overlapping missingness among eligible NHANES 2007–2018 participants; Table S5: Baseline characteristics of participants included in and excluded from the complete-case analysis; Table S6: Omnibus tests for interactions of sitting time, leisure-time physical activity, and CKM stratum with mortality; Table S7: Associations of daily sitting time and leisure-time physical activity with all-cause and heart-disease mortality according to CKM stage group; Table S8: Sensitivity analysis of the joint associations of three leisure-time physical activity categories and three sitting-time categories with all-cause and heart-disease mortality across CKM strata; Table S9: Mutually adjusted associations of daily sitting time and leisure-time physical activity with all-cause and heart-disease mortality across CKM strata.
