# Supplementary material for: Associations of Sitting Time and Leisure-Time Physical Activity with Mortality Across Cardiovascular–Kidney–Metabolic Syndrome Stages
Source: Healthcare (Basel). 2026 Oct 27;14(21):3636. doi: 10.3390/healthcare14213636 (PMC13763375; doi:10.3390/healthcare14213636)
Supplement: Supplementary file 1 [file healthcare-14-03636-s001.zip › healthcare-4538671-supplementary.pdf]

Supplementary Materials

Table S1. Operationalization of cardiovascular–kidney–metabolic stages in NHANES 2007–2018.

Table S2. Characteristics of the study population by leisure-time physical activity level.

Table S3. Participants and all-cause deaths by CKM stage and sex, NHANES 2007–2018.

Table S4. Variable-specific and overlapping missingness among eligible NHANES 2007–2018 participants.

Table S5. Baseline characteristics of participants included in and excluded from the complete-case analysis.

Table S6. Omnibus tests for interactions of sitting time, leisure-time physical activity, and CKM stratum with mortality.

Figure S1. Weighted distribution of joint categories of leisure-time physical activity and daily sitting time among individuals with CKM.

Figure S2. Sex- and age-stratified associations of leisure-time physical activity and sitting time with all-cause mortality.

Figure S3. Associations of daily sitting time with mortality according to leisure-time physical activity among individuals with CKM.

Table S7. Associations of daily sitting time and leisure-time physical activity with all-cause and heart-disease mortality according to CKM stage group (stages 0–2, stage 3, and stage 4).

Table S8. Sensitivity analysis of the joint associations of three leisure-time physical activity categories and three sitting-time categories with all-cause and heart-disease mortality across CKM strata.

Table S9. Mutually adjusted associations of daily sitting time and leisure-time physical activity with all-cause and heart-disease mortality across CKM strata.

Table S1. Operationalization of cardiovascular–kidney–metabolic stages in NHANES 2007–2018.

CKM stage	AHA definition	Operational definition in NHANES 2007–2018	Stage-assignment rule	Participants aged 20–29 years
Stage 0	No CKM risk factors.	Normal BMI and waist circumference; normoglycaemia; normotension; normal triglyceride and HDL-cholesterol profile; low-risk kidney category; no stage 3 risk equivalent; and no clinical CVD.	Assigned only after excluding every higher-stage criterion and confirming all stage-0 components.	Eligible when all measured components were normal.
Stage 1	Excess or dysfunctional adiposity without a higher-stage criterion.	At least one of overweight/obesity, abdominal obesity, or prediabetes, with no stage 2–4 criterion.	Assigned only after excluding stages 2–4.	Eligible on the basis of adiposity or prediabetes.
Stage 2	Metabolic risk factors or moderate-to-high-risk CKD without a higher-stage criterion.	At least one of triglycerides ≥135 mg/dL, hypertension, diabetes, metabolic syndrome, or moderate-to-high-risk CKD, with no stage 3–4 criterion.	Assigned only after excluding stages 3–4.	Eligible on the basis of metabolic or kidney criteria.
Stage 3	Subclinical CVD or a risk equivalent (very-high-risk CKD or high predicted CVD risk) without clinical CVD.	Very-high-risk CKD according to the KDIGO eGFR–UACR matrix or PREVENT-CVD 10-year total-CVD risk ≥20%, in the absence of clinical CVD. Direct subclinical-CVD measures were not consistently available.	Assigned only in the absence of clinical CVD.	PREVENT-CVD was not applied below age 30; stage 3 required very-high-risk CKD. All six participants aged 20–29 years assigned to stage 3 met this kidney criterion.
Stage 4	Clinical CVD overlapping with excess/dysfunctional adiposity, metabolic risk factors, or CKD.	Self-reported physician diagnosis of heart failure, coronary heart disease, myocardial infarction, or stroke together with at least one adiposity, metabolic, or kidney risk factor.	Highest applicable stage.	Eligible when clinical CVD coexisted with a CKM risk factor.

Component criteria

Component	Threshold or operational definition	NHANES variables/source	Role in staging
Overweight/obesity	BMI ≥25 kg/m²; BMI ≥23 kg/m² for non-Hispanic Asian participants when separately identifiable.	BMXBMI; RIDRETH3 for identifiable non-Hispanic Asian ancestry.	Stage 1 adiposity criterion; CKM risk factor for stages 3–4.

Component	Threshold or operational definition	NHANES variables/source	Role in staging
Abdominal obesity	Waist circumference ≥88 cm in women or ≥102 cm in men; ≥80 cm in Asian women or ≥90 cm in Asian men when Asian ancestry was separately identifiable.	BMXWAIST; RIAGENDR; RIDRETH3 when available.	Stage 1 adiposity criterion; CKM risk factor for stages 3–4.
Prediabetes	Fasting plasma glucose 100–124 mg/dL or HbA1c 5.7%–6.4%, without diagnosed or treated diabetes.	LBXGLU; LBXGH; DIQ010; DIQ050; DIQ070.	Stage 1 dysfunctional-adiposity criterion; CKM risk factor for stages 3–4.
Hypertriglyceridaemia	Triglycerides ≥135 mg/dL.	LBXTR.	Stage 2 metabolic criterion.
Hypertension	Mean seated SBP ≥130 mmHg or DBP ≥80 mmHg, self-reported physician diagnosis, or antihypertensive medication use.	BPXSY1–BPXSY4; BPXDI1–BPXDI4; BPQ020; BPQ050A; prescription-medication files.	Stage 2 metabolic criterion.
Diabetes	Fasting plasma glucose ≥126 mg/dL, HbA1c ≥6.5%, self-reported physician diagnosis, insulin use, or oral glucose-lowering medication use.	LBXGLU; LBXGH; DIQ010; DIQ050; DIQ070.	Stage 2 metabolic criterion.
Metabolic syndrome	At least 3 of: abdominal obesity; HDL cholesterol <40 mg/dL in men or <50 mg/dL in women; triglycerides ≥150 mg/dL; SBP ≥130 mmHg, DBP ≥80 mmHg, or antihypertensive treatment; fasting plasma glucose ≥100 mg/dL.	BMXWAIST; RIAGENDR; LBDHDD; LBXTR; BP examination/medication variables; LBXGLU.	Stage 2 metabolic criterion.
eGFR	2021 race-free CKD-EPI creatinine equation: 142 × min(Scr/κ,1)^α × max(Scr/κ,1)^−1.200 × 0.9938^age × 1.012 if female; κ=0.7 (female) or 0.9 (male), α=−0.241 (female) or −0.302 (male).	LBXSCR; RIDAGEYR; RIAGENDR.	Kidney-risk categorization and PREVENT-CVD.
UACR	Urine albumin-to-creatinine ratio in mg/g, calculated from spot urine albumin and urine creatinine.	URXUMA and URXUCR.	Kidney-risk categorization.
Moderate-to-high-risk CKD	KDIGO moderate/high risk: G1–G2 with A2–A3, G3a with A1–A2, or G3b with A1.	Derived eGFR and UACR categories.	Stage 2 kidney criterion.

Component	Threshold or operational definition	NHANES variables/source	Role in staging
Very-high-risk CKD	KDIGO very high risk: G3a with A3, G3b with A2–A3, or G4–G5 with any albuminuria category.	Derived eGFR and UACR categories.	Stage 3 risk equivalent.
High predicted CVD risk	AHA PREVENT base equation for 10-year total CVD risk among adults aged 30–79 years without clinical CVD; high risk was ≥20%.	Required base inputs: sex, age, total cholesterol, HDL cholesterol, SBP, BMI, eGFR, diabetes, current smoking, antihypertensive use, and lipid-lowering therapy. Optional UACR, HbA1c, and social-deprivation inputs were not required for the base equation.	Stage 3 risk equivalent.
Clinical CVD	Self-reported physician diagnosis of heart failure, coronary heart disease, myocardial infarction, or stroke.	MCQ160B, MCQ160C, MCQ160E, and MCQ160F.	Stage 4 criterion.

Notes

Stage assignment rule: Stages were assigned hierarchically from the highest applicable stage (stage 4) downward. A participant satisfying more than one stage was classified in the highest stage.

Missing components: A participant with insufficient required information to determine a stage was left unassigned rather than assumed to have a normal component or stage 0. Participants without an assigned CKM stage were excluded before exposure and covariate complete-case selection.

Age 20–29 years: PREVENT-CVD was not calculated below age 30. Adults aged 20–29 years remained eligible for stages 0–2, stage 3 through very-high-risk CKD, and stage 4 through clinical CVD plus a CKM risk factor.

Stage 3 limitation: Coronary artery calcium/other vascular imaging, echocardiography, NT-proBNP, and high-sensitivity troponin were not consistently available across all cycles. Stage 3 therefore represents an operational approximation using AHA-defined risk equivalents available in NHANES.

Stage 4 limitation: Peripheral artery disease and atrial fibrillation were not consistently available as harmonized clinical-CVD components across all included cycles.

Asian-specific thresholds: Non-Hispanic Asian ancestry was separately identifiable from 2011–2012 onward. General anthropometric thresholds were used in earlier cycles when Asian ancestry could not be separately identified; this data limitation is acknowledged.

Primary grouping: Stages 0–2 were grouped as non-advanced CKM and stages 3–4 as advanced CKM for the prespecified primary stratified analysis. Individual stages were retained in sensitivity analyses.

Source: Ndumele CE et al. Cardiovascular-Kidney-Metabolic Health: A Presidential Advisory From the American Heart Association. Circulation. 2023;148:1606–1635. doi:10.1161/CIR.0000000000001184.

Risk-equation source: Khan SS et al. Novel Prediction Equations for Absolute Risk Assessment of Total Cardiovascular Disease Incorporating Cardiovascular-Kidney-Metabolic Health. Circulation. 2023;148:1982–2004. doi:10.1161/CIR.0000000000001191.

Abbreviations: AHA, American Heart Association; ASCVD, atherosclerotic cardiovascular disease; BMI, body mass index; CKD, chronic kidney disease; CKM, cardiovascular–kidney–metabolic; CVD, cardiovascular disease; DBP, diastolic blood pressure; eGFR, estimated glomerular filtration rate; HbA1c, glycated

hemoglobin; HDL, high-density lipoprotein; KDIGO, Kidney Disease: Improving Global Outcomes; NHANES, National Health and Nutrition Examination Survey; PREVENT, Predicting Risk of Cardiovascular Disease EVENTs; SBP, systolic blood pressure; UACR, urine albumin-to-creatinine ratio.

Table S2. Characteristics of the study population by leisure-time physical activity level.

Characteristic	Overall (n = 15,444)	Inactive (0 min/wk; n = 8,324)	Insufficiently active (>0 to <150 min/wk; n = 2,368)	Active (≥150 min/wk; n = 4,752)	P value
**Sex, n (weighted %)**					**<0.001**
Female	7,683 (49.5%)	4,282 (51.0%)	1,299 (54.0%)	2,102 (45.4%)	
Male	7,761 (50.5%)	4,042 (49.0%)	1,069 (46.0%)	2,650 (54.6%)	
Age, years, weighted mean ± SD	50.0±15.5	52.5±14.9	49.9±15.0	46.8±16.0	<0.001
**Race, n (weighted %)**					**<0.001**
Mexican American	2,322 (7.9%)	1,407 (9.2%)	285 (6.0%)	630 (7.1%)	
Non-Hispanic White	6,402 (68.8%)	3,318 (66.0%)	1,027 (72.2%)	2,057 (70.9%)	
Non-Hispanic Black	3,423 (10.9%)	1,883 (12.0%)	545 (10.4%)	995 (9.8%)	
Other Hispanic	1,574 (5.1%)	950 (5.9%)	213 (4.0%)	411 (4.5%)	
Other race	1,723 (7.2%)	766 (6.9%)	298 (7.4%)	659 (7.7%)	
**PIR, n (weighted %)**					**<0.001**
Low (≤1.39)	5,438 (23.4%)	3,516 (30.1%)	675 (17.8%)	1,247 (16.9%)	
Medium (>1.39 to ≤3.49)	5,325 (33.1%)	2,961 (36.6%)	832 (32.1%)	1,532 (28.7%)	
High (>3.49)	4,681 (43.5%)	1,847 (33.3%)	861 (50.1%)	1,973 (54.4%)	
**Marital status, n (weighted %)**					**<0.001**
Married/cohabiting	9,332 (65.1%)	4,964 (63.9%)	1,471 (67.0%)	2,897 (65.9%)	
Never married	2,402 (15.1%)	1,108 (13.1%)	328 (13.7%)	966 (18.4%)	
Widowed/divorced/separated	3,710 (19.8%)	2,252 (22.9%)	569 (19.3%)	889 (15.7%)	
**Education, n (weighted %)**					**<0.001**
Above high school	8,083 (61.0%)	3,491 (49.6%)	1,436 (67.7%)	3,156 (73.4%)	

Characteristic	Overall (n = 15,444)	Inactive (0 min/wk; n = 8,324)	Insufficiently active (>0 to <150 min/wk; n = 2,368)	Active (≥150 min/wk; n = 4,752)	P value
High school or equivalent	3,559 (23.5%)	2,082 (27.5%)	520 (21.6%)	957 (19.0%)	
Under high school	3,802 (15.5%)	2,751 (22.9%)	412 (10.7%)	639 (7.6%)	
**Smoking status, n (weighted %)**					**<0.001**
Never smoker	8,091 (52.7%)	4,014 (47.3%)	1,319 (56.0%)	2,758 (58.5%)	
Former smoker	4,123 (27.8%)	2,225 (27.6%)	649 (28.5%)	1,249 (27.7%)	
Current smoker	3,230 (19.6%)	2,085 (25.2%)	400 (15.5%)	745 (13.9%)	
**Alcohol consumption, n (weighted %)**					**<0.001**
Never	2,080 (9.9%)	1,274 (11.6%)	312 (9.7%)	494 (7.7%)	
Former	2,658 (13.9%)	1,725 (17.9%)	364 (11.9%)	569 (9.5%)	
Mild	5,333 (38.1%)	2,536 (34.2%)	918 (41.7%)	1,879 (41.8%)	
Moderate	2,361 (17.7%)	1,138 (16.0%)	380 (17.8%)	843 (20.1%)	
Heavy	3,012 (20.3%)	1,651 (20.4%)	394 (18.9%)	967 (20.8%)	
**Daily sitting time, h/d, n (weighted %)**					**0.046**
≤4	6,210 (35.6%)	3,387 (35.8%)	901 (33.5%)	1,922 (36.3%)	
>4 to ≤8	6,130 (40.4%)	3,279 (40.3%)	958 (39.4%)	1,893 (41.0%)	
>8	3,104 (24.0%)	1,658 (23.9%)	509 (27.0%)	937 (22.7%)	
**CKM stage, n (weighted %)**					**<0.001**
Non-advanced (stages 0–2)	12,392 (82.0%)	6,330 (77.3%)	1,972 (85.4%)	4,090 (86.8%)	
Advanced (stages 3–4)	3,052 (18.0%)	1,994 (22.7%)	396 (14.6%)	662 (13.2%)	

Data are unweighted n (survey-weighted %) unless otherwise indicated. Continuous data are presented as survey-weighted mean ± SD. P values were calculated using a design-based Wald test for age and Rao–Scott design-adjusted F tests for categorical variables. CKM, cardiovascular–kidney–metabolic; LTPA, leisure-time physical activity; PIR, poverty income ratio.

Table S3. Participants and all-cause deaths by CKM stage and sex, NHANES 2007–2018.

CKM stage	Male, n/N	Female, n/N	Total, n/N
0	3/198	0/349	3/547
1	26/918	20/1109	46/2027
2	337/4795	269/5023	606/9818
3	186/670	90/375	276/1045
4	344/1180	208/827	552/2007

Values are all-cause deaths/participants (n/N). CKM, cardiovascular–kidney–metabolic; NHANES, National Health and Nutrition Examination Survey.

Table S4. Variable-specific and overlapping missingness among eligible NHANES 2007–2018 participants.

Panel A. Variable-specific missingness

Domain	Variable	Denominator, n	Missing, n	Missing, %	Available, n
CKM	CKM stage	32,016	12,731	39.76	19,285
Behavioral covariate	Alcohol use	32,016	5,002	15.62	27,014
Sociodemographic	Poverty-income ratio	32,016	3,204	10.01	28,812
Mortality	Follow-up time	32,016	1,232	3.85	30,784
Exposure	Original sitting category	32,016	181	0.57	31,835
Exposure	Daily sitting time	32,016	181	0.57	31,835
Mortality	All-cause death indicator	32,016	113	0.35	31,903
Mortality	Heart-disease death indicator	32,016	113	0.35	31,903
Mortality	Mortality linkage status	32,016	113	0.35	31,903

Domain	Variable	Denominator, n	Missing, n	Missing, %	Available, n
Sociodemographic	Education	32,016	36	0.11	31,980
Exposure	LTPA minutes	32,016	34	0.11	31,982
Exposure	LTPA category	32,016	34	0.11	31,982
Sociodemographic	Marital status	32,016	22	0.07	31,994
Behavioral covariate	Smoking	32,016	20	0.06	31,996
Sociodemographic	Age	32,016	0	0.00	32,016
Sociodemographic	Race/ethnicity	32,016	0	0.00	32,016
Sociodemographic	Sex	32,016	0	0.00	32,016
Survey design	Primary sampling unit	32,016	0	0.00	32,016
Survey design	Survey stratum	32,016	0	0.00	32,016
Survey design	MEC examination weight	32,016	0	0.00	32,016

Panel B. Overlapping missingness patterns

Missingness pattern	n	%
Complete	15,444	48.24
CKM	9,026	28.19
Covariates	3,584	11.19
CKM + Covariates	2,556	7.98
CKM + Mortality + Covariates	1,055	3.30
Mortality + Covariates	122	0.38
Exposure	72	0.22
Exposure + Covariates	47	0.15
CKM + Exposure	33	0.10
CKM + Mortality	23	0.07
CKM + Exposure + Covariates	22	0.07
CKM + Exposure + Mortality + Covariates	16	0.05
Mortality	14	0.04
Exposure + Mortality + Covariates	2	0.01

Notes: The denominator for missingness summaries was 32,016 nonpregnant adults aged 20-79 years. Exposure missingness indicates any missing LTPA or sitting-time measure; mortality missingness indicates any missing mortality linkage status, follow-up time, or death indicator; covariate missingness indicates any missing marital status, poverty-income ratio, education, smoking, or alcohol-use value. Counts across variables and domains overlap and should not be summed. CKM, cardiovascular-kidney-metabolic; LTPA, leisure-time physical activity.

Table S5. Baseline characteristics of participants included in and excluded from the complete-case analysis

Characteristic	Level	Included (n=15,444)	Excluded (n=16,572)	Absolute SMD	Design-based P
**Age, years**		**50.05 (15.54)**	**42.32 (15.12)**	**0.504**	**<0.001**
**Sex**				**0.052**	**<0.001**
	Male	7761 (50.5%)	7989 (47.9%)	0.052	
	Female	7683 (49.5%)	8583 (52.1%)	0.052	
**Race/ethnicity**				**0.150**	**<0.001**
	Mexican American	2322 (7.9%)	2686 (9.7%)	0.062	
	Other Hispanic	1574 (5.1%)	1930 (7.0%)	0.079	
	Non-Hispanic White	6402 (68.8%)	5865 (61.6%)	0.150	
	Non-Hispanic Black	3423 (10.9%)	3656 (12.2%)	0.039	
	Other Race - Including Multi-Racial	1723 (7.2%)	2435 (9.5%)	0.080	
**Education**				**0.030**	**0.203**
	Under high school	3802 (15.5%)	4042 (15.8%)	0.009	
	High school or equivalent	3559 (23.5%)	3696 (22.3%)	0.030	
	Above high school	8083 (61.0%)	8798 (61.9%)	0.019	
**Poverty–income ratio**				**0.047**	**0.022**
	Low(≤1.39)	5438 (23.4%)	4876 (25.4%)	0.047	

Characteristic	Level	Included (n=15,444)	Excluded (n=16,572)	Absolute SMD	Design-based P
	Medium(>1.39,<=3.49)	5325 (33.1%)	4454 (32.0%)	0.024	
	High(>3.49)	4681 (43.5%)	4038 (42.6%)	0.019	
**Marital status**				**0.215**	**<0.001**
	Married/cohabiting	9332 (65.1%)	9758 (62.1%)	0.063	
	Widowed/divorced/separated	3710 (19.8%)	2901 (14.4%)	0.144	
	Never married	2402 (15.1%)	3891 (23.6%)	0.215	
**Smoking**				**0.189**	**<0.001**
	Never smoker	8091 (52.7%)	9782 (58.6%)	0.120	
	Former smoker	4123 (27.8%)	3086 (19.7%)	0.189	
	Current smoker	3230 (19.6%)	3684 (21.7%)	0.052	
**Alcohol use**				**0.123**	**<0.001**
	never	2080 (9.9%)	1715 (11.3%)	0.046	
	former	2658 (13.9%)	1385 (10.0%)	0.123	
	mild	5333 (38.1%)	3707 (34.9%)	0.067	
	moderate	2361 (17.7%)	1981 (18.6%)	0.021	
	heavy	3012 (20.3%)	2782 (25.2%)	0.118	
**CKM stage**				**0.084**	**0.008**
	Stage 0	547 (4.8%)	110 (4.2%)	0.028	

Characteristic	Level	Included (n=15,444)	Excluded (n=16,572)	Absolute SMD	Design-based P
	Stage 1	2027 (15.6%)	456 (13.0%)	0.074	
	Stage 2	9818 (61.6%)	2381 (61.7%)	0.003	
	Stage 3	1045 (7.5%)	212 (7.8%)	0.010	
	Stage 4	2007 (10.5%)	682 (13.3%)	0.084	
**LTPA, min/week**		**195.33 (366.51)**	**251.14 (422.50)**	**0.141**	**<0.001**
**Sitting time, h/day**		**6.33 (3.37)**	**5.99 (3.42)**	**0.099**	**<0.001**

Notes: The source population comprised 32,016 NHANES 2007–2018 participants aged 20–79 years after excluding pregnant participants; 15,444 complete cases were included in the primary analysis. Continuous variables are presented as survey-weighted mean (SD), and categorical variables as unweighted n (survey-weighted %), calculated among participants with observed values for the corresponding characteristic. SMD denotes the absolute standardized mean difference; for categorical variables, the characteristic-level row reports the maximum category-specific SMD. P values were obtained using survey-weighted t tests for continuous variables and Rao–Scott design-adjusted F tests for categorical variables. Survey analyses incorporated SDMVSTRA, SDMVPSU, and the six-cycle MEC examination weight (WTMEC2YR/6), with variance estimation by Taylor-series linearization. A total of 1,144 eligible participants had WTMEC2YR = 0; all were in the excluded group and therefore contributed to unweighted counts but not to MEC-weighted estimates or design-based tests. CKM, cardiovascular–kidney–metabolic; LTPA, leisure-time physical activity; MEC, mobile examination center; PSU, primary sampling unit; SD, standard deviation; SMD, standardized mean difference.

Table S6. Omnibus tests for interactions of sitting time, leisure-time physical activity, and CKM stratum with mortality.

Outcome	Interaction	Population	Events	F statistic	Numerator df	Denominator df	P value
All-cause mortality	CKM × sitting time (3 × 2; omnibus)	Overall	1,483	2.556	2	70	0.085
All-cause mortality	CKM × LTPA (2 × 3; omnibus)	Overall	1,483	0.154	2	70	0.858
All-cause mortality	Joint sitting-time/LTPA category × CKM stratum (omnibus)	Overall	1,483	1.022	5	66	0.412
All-cause mortality	Sitting time × LTPA (3 × 3; omnibus)	Overall	1,483	1.285	4	68	0.285
All-cause mortality	Sitting time × LTPA (3 × 3; omnibus)	Non-advanced CKM	655	2.498	4	69	0.051
All-cause mortality	Sitting time × LTPA (3 × 3; omnibus)	Advanced CKM	828	1.913	4	69	0.118
Heart-disease mortality	CKM × sitting time (3 × 2; omnibus)	Overall	354	2.049	2	70	0.137
Heart-disease mortality	CKM × LTPA (2 × 3; omnibus)	Overall	354	0.333	2	70	0.718
Heart-disease mortality	Joint sitting-time/LTPA category × CKM stratum (omnibus)	Overall	354	1.349	5	66	0.255
Heart-disease mortality	Sitting time × LTPA (3 × 3; omnibus)	Overall	354	0.279	4	68	0.890
Heart-disease mortality	Sitting time × LTPA (3 × 3; omnibus)	Non-advanced CKM	126	0.910	4	69	0.463
Heart-disease mortality	Sitting time × LTPA (3 × 3; omnibus)	Advanced CKM	228	1.019	4	69	0.404

Notes: Omnibus interactions were evaluated using design-based Wald F tests from survey-weighted Cox proportional hazards models incorporating NHANES strata, primary sampling units, and the six-cycle MEC examination weight. Models were adjusted for age, sex, race/ethnicity, education, poverty-income ratio, marital status, smoking, and alcohol use. CKM, cardiovascular–kidney–metabolic; LTPA, leisure-time physical activity; MEC, mobile examination center.

Figure S1. Weighted distribution of joint categories of leisure-time physical activity and daily sitting time among individuals with CKM.

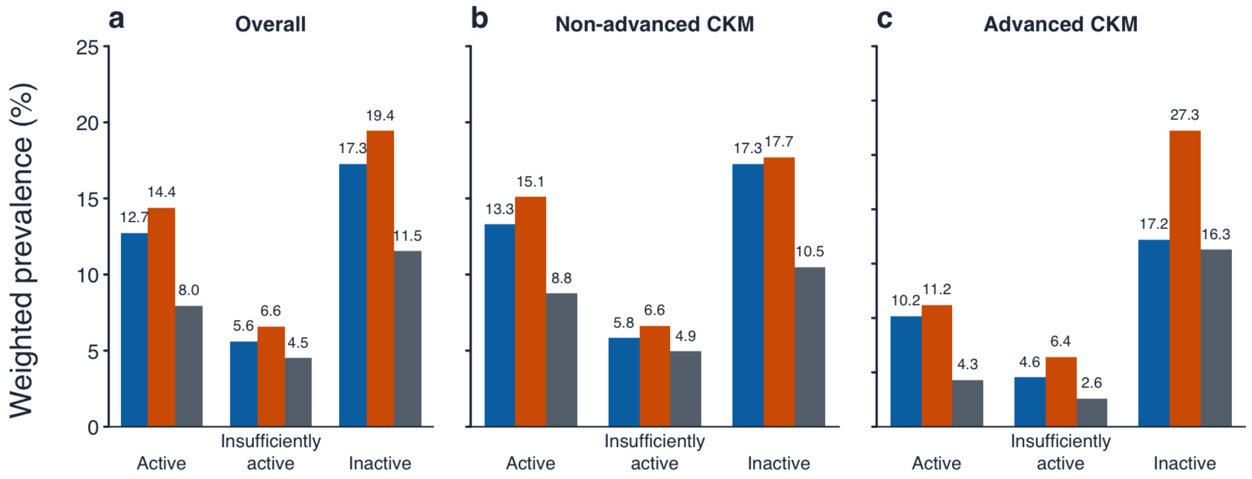

Survey-weighted percentages are shown for the overall CKM population (a), non-advanced CKM (b), and advanced CKM (c). Leisure-time physical activity was categorized as inactive (0 min/wk), insufficiently active (>0 to <150 min/week), or active (≥150 min/week). Blue, orange, and gray bars indicate ≤4, >4 to ≤8, and >8 h/day of sitting, respectively.

Figure S2. Sex- and age-stratified joint associations of leisure-time physical activity and sitting time with all-cause mortality.

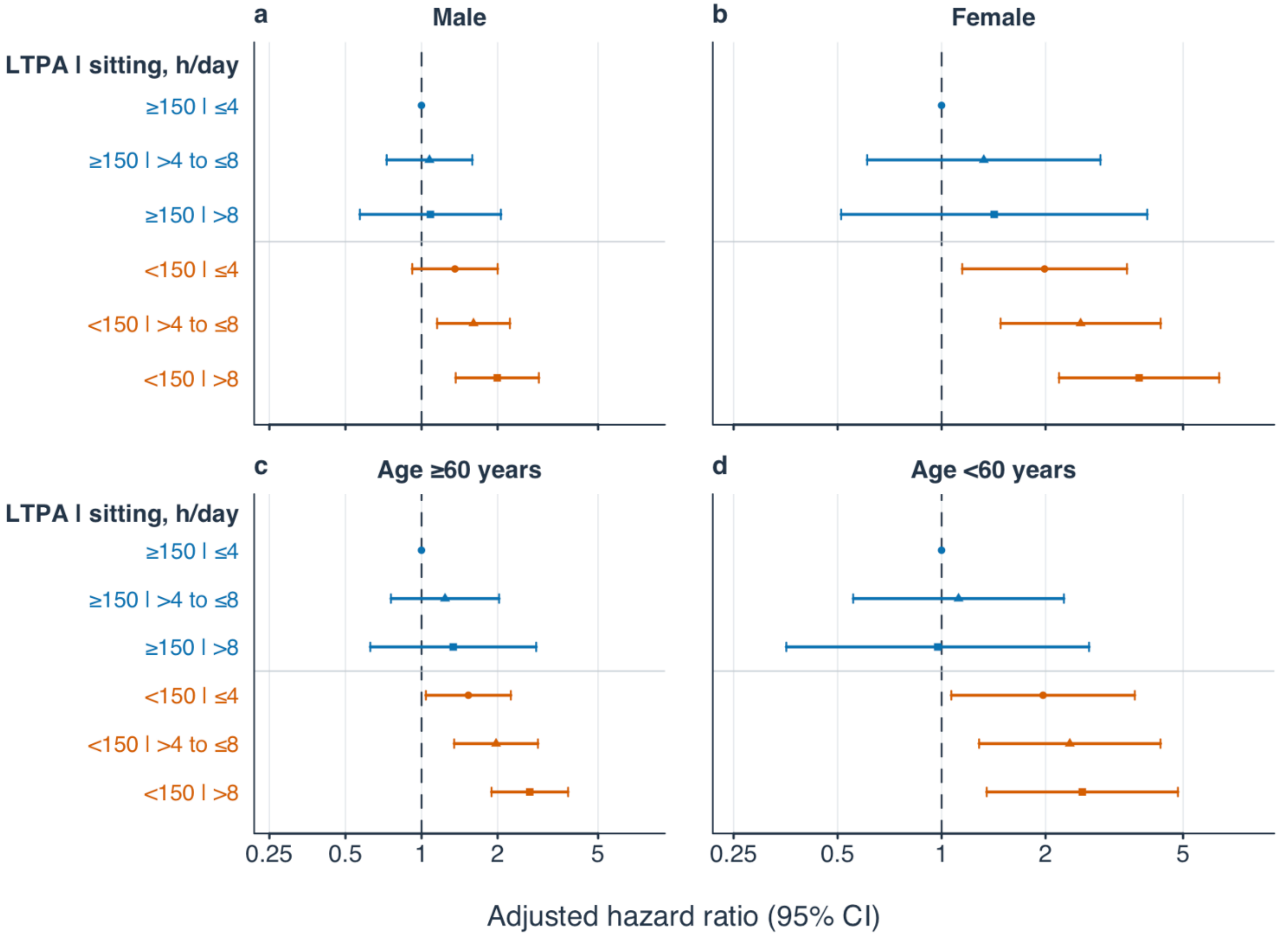

Points represent adjusted hazard ratios and horizontal lines represent 95% confidence intervals. Reference category: leisure-time physical activity ≥150 min/week and daily sitting time ≤4 h/day. Models were adjusted for age, sex, race/ethnicity, education, poverty income ratio, marital status, smoking status, and alcohol consumption, except for the corresponding stratification variable. **Abbreviations**: CKM, cardiovascular–kidney–metabolic; LTPA, leisure-time physical activity.

Figure S3. Associations of daily sitting time with mortality according to leisure-time physical activity among individuals with CKM.

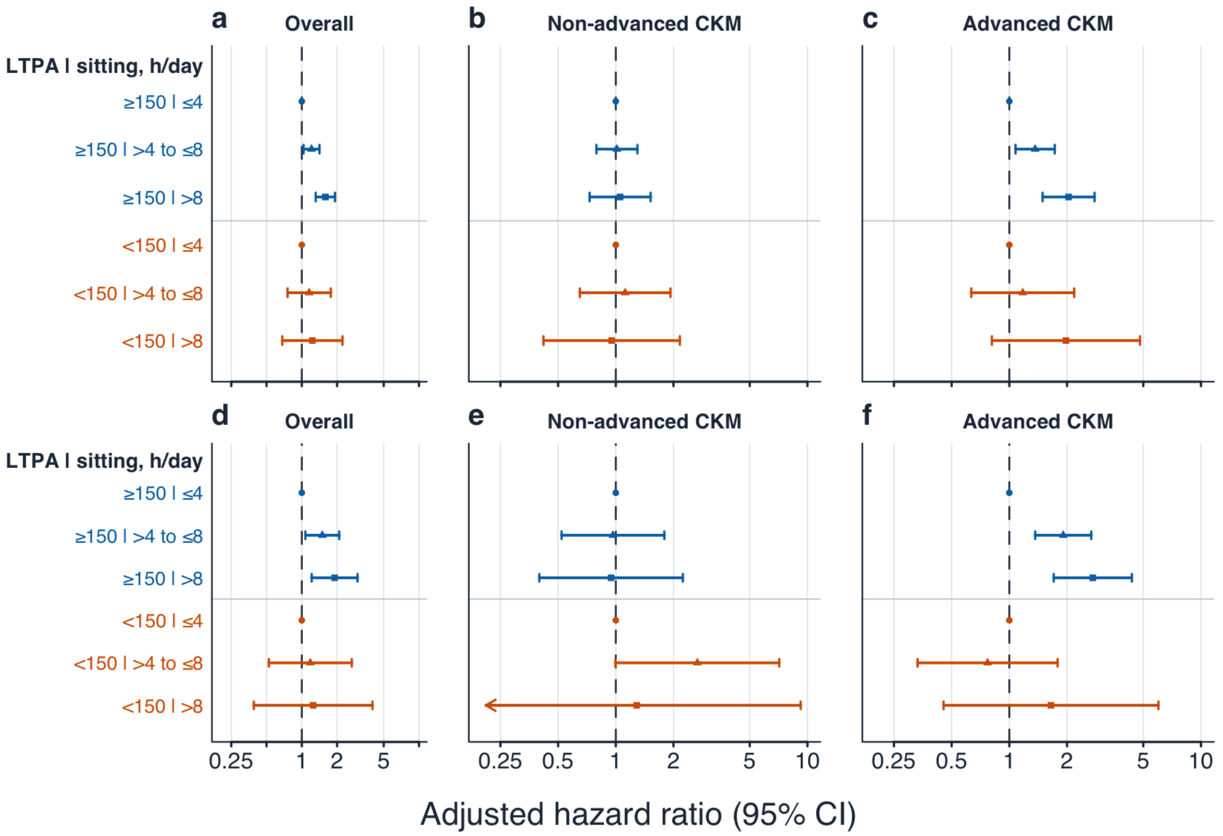

Panels a–c show all-cause mortality and panels d–f show heart-disease mortality in the overall CKM population, non-advanced CKM, and advanced CKM, respectively. Points represent adjusted hazard ratios and horizontal lines represent 95% confidence intervals. Reference category within each leisure-time physical activity stratum: ≤4 h/day of sitting. Models were adjusted for age, sex, race/ethnicity, education, poverty income ratio, marital status, smoking status, and alcohol consumption. Arrows indicate confidence intervals extending beyond the plotted range.

Table S7. Associations of daily sitting time and leisure-time physical activity with all-cause and heart-disease mortality according to CKM stage group (stages 0–2, stage 3, and stage 4).

Exposure	CKM stages 0–2		CKM stage 3		CKM stage 4	
	Deaths/N	HR (95% CI)	Deaths/N	HR (95% CI)	Deaths/N	HR (95% CI)
**All-cause mortality**						
**Daily sitting time, h/day**						
≤4	282/5176	1.00 [Reference]	89/369	1.00 [Reference]	150/665	1.00 [Reference]
>4 to ≤8	260/4757	1.04 (0.82–1.32)	118/467	1.05 (0.77–1.42)	269/906	1.65 (1.30–2.08)
>8	113/2459	1.07 (0.76–1.51)	69/209	2.24 (1.56–3.22)	133/436	1.91 (1.38–2.65)
**LTPA, min/week**						
Inactive	455/6330	1.00 [Reference]	190/626	1.00 [Reference]	438/1368	1.00 [Reference]
Insufficiently active	80/1972	0.72 (0.53–0.97)	32/149	0.46 (0.30–0.70)	53/247	0.73 (0.55–0.97)
Active	120/4090	0.58 (0.42–0.81)	54/270	0.61 (0.38–0.97)	61/392	0.45 (0.30–0.67)
**Heart-disease mortality**						
**Daily sitting time, h/day**						
≤4	54/5176	1.00 [Reference]	14/369	1.00 [Reference]	46/665	1.00 [Reference]
>4 to ≤8	56/4757	1.12 (0.64–1.94)	31/467	1.39 (0.66–2.92)	83/906	1.94 (1.38–2.74)
>8	16/2459	1.03 (0.46–2.30)	18/209	3.46 (1.41–8.49)	36/436	2.03 (1.25–3.29)
**LTPA, min/week**						
Inactive	93/6330	1.00 [Reference]	48/626	1.00 [Reference]	132/1368	1.00 [Reference]
Insufficiently active	11/1972	0.51 (0.22–1.20)	7/149	0.32 (0.11–0.87)	13/247	0.70 (0.33–1.49)
Active	22/4090	0.41 (0.21–0.80)	8/270	0.38 (0.10–1.51)	20/392	0.55 (0.27–1.13)

Notes: Values are deaths/N and hazard ratios (HRs) with 95% confidence intervals (CIs). Stage-specific sensitivity analyses used the same covariate adjustment as the primary survey-weighted Cox models. Reference categories were ≤4 h/day for sitting time and inactive LTPA (0 min/week). CKM, cardiovascular–kidney–metabolic; CI, confidence interval; HR, hazard ratio; LTPA, leisure-time physical activity.

Table S8. Sensitivity analysis of the joint associations of three leisure-time physical activity categories and three sitting-time categories with all-cause and heart-disease mortality across CKM strata.

LTPA, min/week	Sitting, h/day	Non-advanced CKM (stages 0–2)		Advanced CKM (stages 3–4)	
		Deaths/N	Adjusted HR (95% CI)	Deaths/N	Adjusted HR (95% CI)
**All-cause mortality**					
Active (≥150)	≤4	51/1,638	1.00 (reference)	47/284	1.00 (reference)
Active (≥150)	>4 to ≤8	56/1,619	1.08 (0.63–1.85)	54/274	1.20 (0.70–2.07)
Active (≥150)	>8	13/833	0.92 (0.42–2.02)	14/104	1.50 (0.66–3.41)
Insufficiently active (>0 to <150)	≤4	35/764	0.91 (0.55–1.50)	35/137	1.51 (0.78–2.92)
Insufficiently active (>0 to <150)	>4 to ≤8	34/772	1.87 (1.07–3.26)	35/186	1.19 (0.64–2.22)
Insufficiently active (>0 to <150)	>8	11/436	0.90 (0.35–2.31)	15/73	1.44 (0.65–3.20)
Inactive (0)	≤4	196/2,774	1.82 (1.16–2.85)	157/613	1.48 (0.89–2.47)
Inactive (0)	>4 to ≤8	170/2,366	1.59 (1.02–2.49)	298/913	2.24 (1.39–3.58)
Inactive (0)	>8	89/1,190	1.93 (1.17–3.20)	173/468	3.27 (2.06–5.19)
**Heart-disease mortality**					
Active (≥150)	≤4	8/1,638	1.00 (reference)	13/284	1.00 (reference)
Active (≥150)	>4 to ≤8	13/1,619	1.91 (0.68–5.39)	12/274	0.82 (0.33–2.04)
Active (≥150)	>8	1/833	0.76 (0.09–6.20)	3/104	1.32 (0.31–5.51)
Insufficiently active (>0 to <150)	≤4	4/764	0.91 (0.24–3.44)	8/137	0.73 (0.17–3.11)
Insufficiently active (>0 to <150)	>4 to ≤8	5/772	1.30 (0.36–4.69)	9/186	1.29 (0.37–4.49)
Insufficiently active (>0 to <150)	>8	2/436	4.05 (0.82–20.00)	3/73	1.04 (0.25–4.40)
Inactive (0)	≤4	42/2,774	3.42 (1.24–9.42)	39/613	1.16 (0.43–3.07)
Inactive (0)	>4 to ≤8	38/2,366	3.26 (1.21–8.77)	93/913	2.24 (0.91–5.55)

LTPA, min/week	Sitting, h/day	Non-advanced CKM (stages 0–2)		Advanced CKM (stages 3–4)	
		Deaths/N	Adjusted HR (95% CI)	Deaths/N	Adjusted HR (95% CI)
Inactive (0)	>8	13/1,190	2.59 (0.95–7.07)	48/468	3.18 (1.22–8.28)

Notes: LTPA was expressed as moderate-intensity-equivalent minutes per week. Adjusted HRs (95% CIs) were estimated using the same covariate adjustment as in the primary survey-weighted Cox models. Within each CKM stratum, the reference group was participants reporting LTPA ≥150 min/week and sitting ≤4 h/day. Heart-disease mortality estimates were based on fewer events and should be interpreted cautiously when confidence intervals were wide. CKM, cardiovascular–kidney–metabolic; CI, confidence interval; HR, hazard ratio; LTPA, leisure-time physical activity.

Table S9. Mutually adjusted associations of daily sitting time and leisure-time physical activity with all-cause and heart-disease mortality across CKM strata.

Exposure	Overall		Non-advanced CKM (stages 0–2)		Advanced CKM (stages 3–4)	
	Deaths/N	Adjusted HR (95% CI)	Deaths/N	Adjusted HR (95% CI)	Deaths/N	Adjusted HR (95% CI)
**All-cause mortality**						
**Daily sitting time, h/day**						
≤4	521/6,210	1.00 [Reference]	282/5,176	1.00 [Reference]	239/1,034	1.00 [Reference]
>4 to ≤8	647/6,130	1.20 (1.03–1.39)	260/4,757	1.03 (0.81–1.30)	387/1,373	1.34 (1.10–1.63)
>8	315/3,104	1.50 (1.28–1.77)	113/2,459	1.03 (0.73–1.46)	202/645	1.92 (1.48–2.51)
**LTPA, min/week**						
Inactive (0)	1,083/8,324	1.00 [Reference]	455/6,330	1.00 [Reference]	628/1,994	1.00 [Reference]
Insufficiently active (>0 to <150)	165/2,368	0.65 (0.53–0.79)	80/1,972	0.72 (0.53–0.97)	85/396	0.63 (0.48–0.82)
Active (≥150)	235/4,752	0.55 (0.44–0.69)	120/4,090	0.58 (0.42–0.81)	115/662	0.56 (0.40–0.77)
**Heart-disease mortality**						
**Daily sitting time, h/day**						

Exposure	Overall		Non-advanced CKM (stages 0–2)		Advanced CKM (stages 3–4)	
	Deaths/N	Adjusted HR (95% CI)	Deaths/N	Adjusted HR (95% CI)	Deaths/N	Adjusted HR (95% CI)
≤4	114/6,210	1.00 [Reference]	54/5,176	1.00 [Reference]	60/1,034	1.00 [Reference]
>4 to ≤8	170/6,130	1.42 (1.05–1.92)	56/4,757	1.09 (0.63–1.91)	114/1,373	1.62 (1.20–2.20)
>8	70/3,104	1.74 (1.14–2.68)	16/2,459	0.97 (0.43–2.20)	54/645	2.28 (1.48–3.51)
**LTPA, min/week**						
Inactive (0)	273/8,324	1.00 [Reference]	93/6,330	1.00 [Reference]	180/1,994	1.00 [Reference]
Insufficiently active (>0 to <150)	31/2,368	0.49 (0.34–0.72)	11/1,972	0.51 (0.22–1.21)	20/396	0.52 (0.29–0.96)
Active (≥150)	50/4,752	0.44 (0.26–0.72)	22/4,090	0.41 (0.21–0.80)	28/662	0.50 (0.26–0.95)

Notes: Values are deaths/N and adjusted hazard ratios (HRs) with 95% confidence intervals (CIs). Sitting-time models were additionally adjusted for LTPA, and LTPA models were additionally adjusted for sitting time, in addition to age, sex, race/ethnicity, education, poverty-income ratio, marital status, smoking, and alcohol use. Reference categories were ≤4 h/day for sitting time and inactive LTPA (0 min/week). LTPA was expressed as moderate-intensity-equivalent minutes per week. CKM, cardiovascular–kidney–metabolic; CI, confidence interval; HR, hazard ratio; LTPA, leisure-time physical activity.
